# The preoperative value of fine‐needle aspiration in adult soft tissue lesions: An analysis of 514 cases at Shanghai Cancer Center

**DOI:** 10.1002/cam4.5156

**Published:** 2022-08-19

**Authors:** Meng Fang, Bingnan Wang, Biqiang Zheng, Wangjun Yan

**Affiliations:** ^1^ Department of Musculoskeletal Oncology Fudan University Shanghai Cancer Center Shanghai China; ^2^ Department of Oncology Shanghai Medical College, Fudan University Shanghai China

**Keywords:** biopsy, fine‐needle aspiration, positive predictive value, soft tissue lesions, soft tissue sarcomas

## Abstract

**Background:**

Fine‐needle aspiration (FNA) cytology is a rapid, inexpensive, and uncomplicated method. However, its role in the assessment of soft tissue lesions (STL) remains controversial, and its ability to guide surgical treatment remains unclear. This study investigated the positive predictive value (PPV) of FNA for detecting malignancy and its guiding role in the surgical treatment of STL.

**Methods:**

We retrospectively reviewed 514 patients with STL who underwent preoperative FNA and surgical resection between March 2015 and August 2021. Imaging assessments confirmed that radical surgery was possible. The FNA results were compared with the final postoperative histopathology.

**Results:**

Of the 514 patients with STL, 496 (mean age, 48.9 years; range, 21–91 years) were eligible for analysis, the male to female ratio was 111:100. According to the 496 FNA results, 90 (18.2%) were positive for malignancy, 84 (16.9%) were suspicious for malignancy, 80 (16.1%) were spindle cell present, and 242 (48.8%) were negative for malignant cells. Compared with postoperative histopathology, FNA correctly detected all 90 malignant lesions and 203 of the 242 benign lesions. A total of 39 false‐negative results were obtained. FNA showed an accuracy of 88.3%, sensitivity of 69.8%, specificity of 100%, negative predictive value (NPV) of 83.9%, and PPV of 100%. In the other seven validation cohorts (*n* = 1157), FNA had a consistently high PPV, with values all more than 93%.

**Conclusion:**

Our results demonstrate that FNA has a high PPV for detecting malignancy. For patients with resectable lesions and malignant FNA, the core needle biopsy (CNB) step can be omitted with multidisciplinary evaluation, and subsequent radical surgery can be performed.

## Graphical abstract

Fine‐needle aspiration (FNA) has a high positive predictive value (PPV) for detecting malignancy in the soft tissue lesions. For patients with resectable lesions and malignant FNA, the core needle biopsy (CNB) step can be omitted with multidisciplinary evaluation, and subsequent radical surgery can be performed.

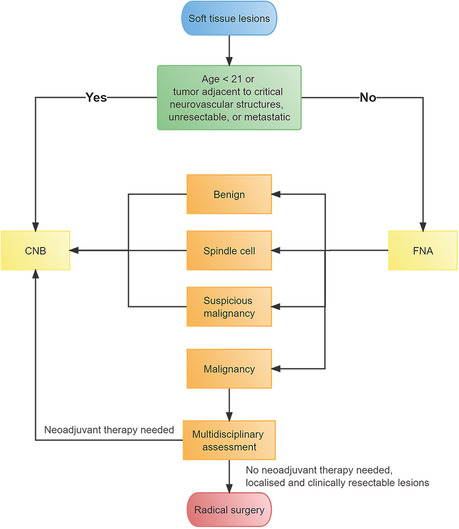

## 1 INTRODUCTION

Soft tissue sarcomas (STS) are rare malignancies, accounting for approximately 1% of adult cancers.^1^ A biopsy is a key step in the diagnosis of musculoskeletal tumors. Biopsy types include fine‐needle aspiration (FNA), core needle biopsy (CNB), incisional biopsy, and excisional biopsy.^2^ Open surgical incision allows obtaining a large tumor sample and provides high diagnostic accuracy; however, it is more invasive and is more likely to cause more spread of the lesion.^3^ Compared with incision biopsy, core needle biopsy is a simple, safe, and less invasive diagnostic method that has been widely used in the preoperative diagnosis of STS.^4^ Many studies from multiple institutions have consistently shown a high diagnostic accuracy for CNB.^5^, ^6^, ^7^ The National Comprehensive Cancer Network (NCCN) guidelines support the utility of CNB for the preoperative diagnosis of STS.^8^

The usefulness of FNA for the diagnosis of soft tissue tumors is controversial. It has long been considered inadequate as a first‐line approach for the diagnosis of STS, and some pathologists are reluctant to endorse this technique because of its limitations, such as cell dispersion, loss of tissue pattern, paucity of the specimen, and inapplicability of histopathologic classification.^9^, ^10^, ^11^ However, compared with CNB, FNA has the advantages of being minimally invasive, well tolerated, has low cost, provides rapid diagnosis, and does not require local anesthesia.^12^, ^13^ Some studies support that FNA is valuable for the initial diagnostic evaluation of soft tissue tumors.^14^, ^15^ However, its capacity to guide the surgical treatment of soft tissue lesions (STL) remains unclear. In this study, to explore the preoperative value of FNA in the management of STL, we compared the results of preoperative FNA with those of final postoperative histopathology. Interestingly, we found that FNA has a unique advantage in determining malignancy, with a 100% positive predictive value (PPV), which is consistent with seven previous validation cohorts (all PPV more than 93%). Surgical resection (with appropriately negative margins) is the standard primary treatment for most patients with STS according to the NCCN Guidelines.^8^ For patients with resectable lesions and malignant FNA, the CNB step can be omitted with a multidisciplinary evaluation, and radical surgery can be performed.

## 2 PATIENTS AND METHODS

### 2.1 Patients

We retrospectively reviewed 514 patients with STL who underwent preoperative FNA and surgical treatment at the Fudan University Shanghai Cancer Center between March 2015 and August 2021. We excluded 9 patients who were younger than 20 years of age and 9 patients whose tumors were adjacent to critical structures (major vessels or vital nerves). Of the 514 patients with soft tissue lesions, 496 were eligible for analysis. This study was reviewed and approved by the institutional review board and ethics committee before the initiation of the study. Informed consent was obtained from all patients.

### 2.2 Methods

For the procedure, each patient was in a sitting, supine, or prone position and had undergone routine disinfection. The FNA procedures were performed with a 22–24‐gauge needle attached to a disposable, 20‐ml syringe for percutaneous puncture of the tumor. Multiple samples were taken from various locations within the tumor to obtain a representative sample. FNA biopsies of patients with deep lesions were performed with ultrasound guidance. After needling, the aspirates were immediately fixed in 95% ethanol and stained with hematoxylin–eosin staining. Cytological diagnoses were classified into four categories: (1) positive for malignant cells, (2) suspicious for malignant cells, (3) spindle cells, and (4) negative for malignant cells (benign).

In our study, verification of the cytopathologic diagnosis was performed through postoperative histopathology. The diagnostic accuracy, sensitivity, specificity, PPV, negative predictive value (NPV), and Youden index were calculated. Diagnostic accuracy was calculated as (correct/[correct + incorrect]), sensitivity was calculated as (true positive/[true positive + false negative]), specificity was calculated as (true negative/[true negative + false positive]), PPV was calculated as (true positive/[true positive + false positive]), NPV was calculated as (true negative/[true negative + false negative]), and the Youden index was calculated as (sensitivity + specificity − 1). To calculate sensitivity, specificity, and PPV, FNA with suspicious (*n* = 84) or spindle cells (*n* = 80) was excluded from the analysis,^16^, ^17^ the results were based on 332 remaining cases.

## 3 RESULT

**FIGURE 1:**
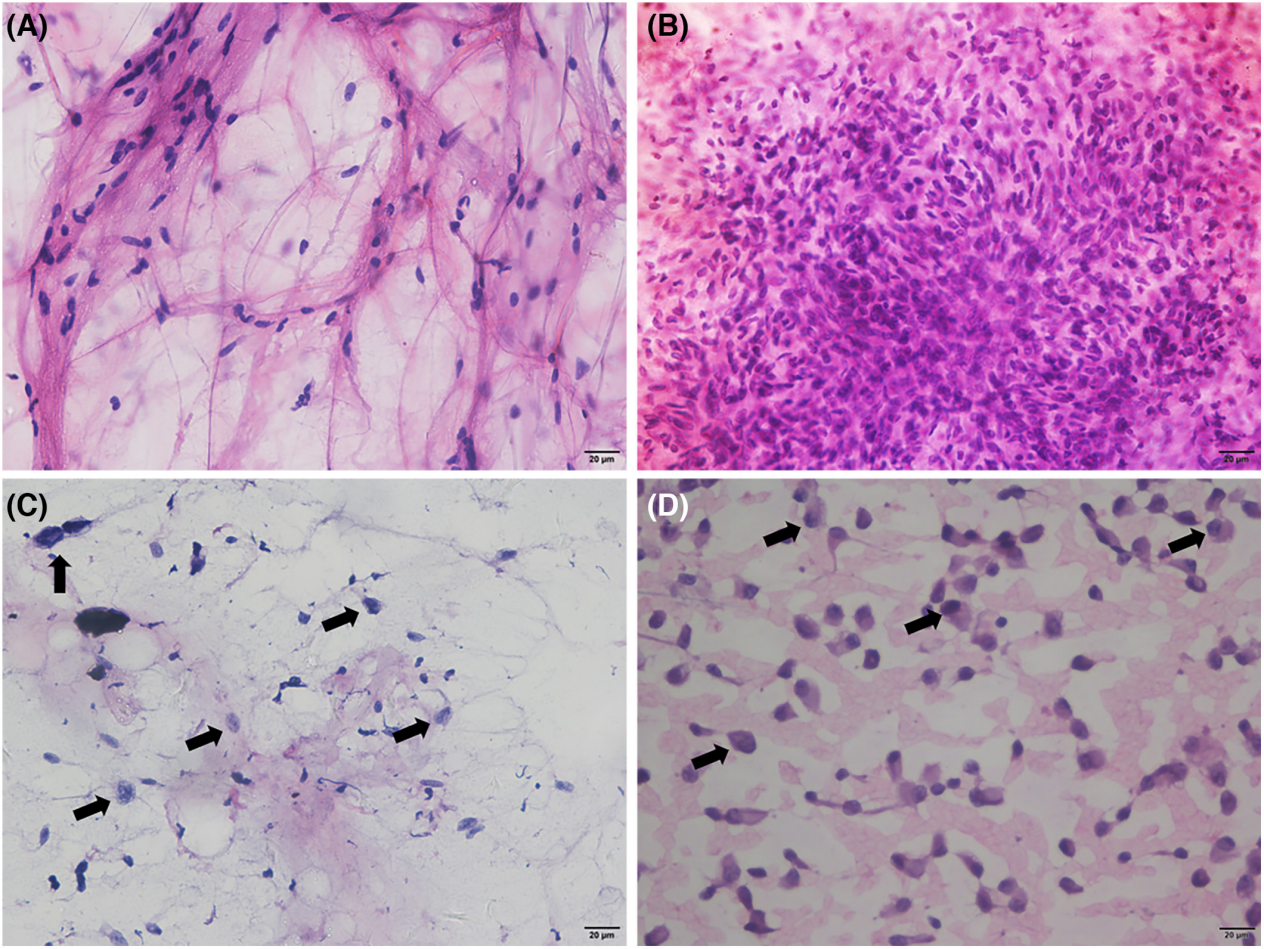
Cytological diagnoses of soft tissue lesions. (A) Adipocytes, negative for malignant cells, the case was diagnosed with lipoma; (B) Spindle cells, the patient was diagnosed with dermatofibrosarcoma protuberans; (C) Atypical cells (arrows), suspicious for malignant cells, the case was diagnosed with myxofibrosarcoma; (D) Positive for malignant cells, the patient was diagnosed with clear cell sarcoma.

To explore the preoperative value of FNA, we compared the results of preoperative FNA with those of the final postoperative histopathology. A total of 514 patients with STL were included in this study between March 2015 and August 2021. Among them, 9 patients aged <20 years and 9 patients with tumors adjacent to critical neurovascular structures were excluded from this study. The reasons for this were as follows: (1) FNA is a minimally invasive and rapid method to detect malignancy but is poor at identifying histological types in STS compared with CNB. (2) Rhabdomyosarcoma, Ewing sarcoma, and other pediatric sarcoma are prevalent in young patients less than 20 years of age. Multidisciplinary evaluation of these sarcomas, including the need for chemotherapy, surgery, and radiation, is strongly encouraged.^8^ Therefore, CNB is more appropriate for determining the pathological types of patients less than 20 years of age. (3) For tumors adjacent to critical vessels or nerves, patients may need neoadjuvant therapy to shrink the tumor volume to preserve neurological or vascular function, and the histological type is indispensable before treatment. A total of 496 patients with STL underwent FNA and surgical resection, including 261 men and 235 women. The male to female ratio was 111:100. Soft tissue lesions were most common in the extremities (*n* = 312), followed by the trunk (*n* = 172), and neck (*n* = 12) (Table 1). The most common malignant pathological types were undifferentiated pleomorphic sarcoma (UPS; *n* = 48), myxofibrosarcoma (*n* = 31), and liposarcoma (*n* = 24). The most common benign lesions were lipomas (*n* = 41), fibromatoses (*n* = 37), and schwannomas (*n* = 33) (Table 1). No complications were associated with FNA, and all patients were discharged from the hospital on the same day. Of the 496 FNA aspirates, 90 (18.2%) were positive for malignancy, 84 (16.9%) were suspicious for malignancy, 80 (16.1%) were spindle cell present, and 242 (48.8%) were negative for malignant cells (Table 1). The representative images of cytological diagnoses were shown in Figure 1. Compared with postoperative pathology, the diagnostic errors for FNA are listed in Table 2. A total of 39 malignant soft tissue tumors were misdiagnosed as benign based on FNA. We found that myxofibrosarcoma and UPS were the most likely to be misdiagnosed. Of these, 11 patients with myxofibrosarcoma were diagnosed with innocent tumors and 11 cases of UPS were misdiagnosed as benign masses based on FNA.

**TABLE 1:** Clinicopathological characteristics of soft tissue lesions

Characteristics	No.	%
Total cases	496	
Age		
21–60	338	68.1
>60	158	31
Average age	48.9 (21–91)	
Gender		
Female	235	47.4
Male	261	52.6
Anatomical location		
Neck	12	2.4
Trunk	172	34.7
Limbs	312	62.9
Primary FNAC interpretations		
Positive for malignancy	90	18.2
Suspicious for malignancy	84	16.9
Spindle cell present	80	16.1
Negative for malignant cells	242	48.8
Paraffin section interpretations		
Malignancies		
UPS	48	9.7
Myxofibrosarcoma	31	6.4
Liposarcoma	24	4.8
Fibrosarcoma	21	4.2
Synovial sarcoma	19	3.8
Leiomyosarcoma	10	2.0
Malignant peripheral nerve sheath tumor	7	1.4
Epithelioid sarcoma	7	1.4
Chondrosarcoma	7	1.4
Rhabdomyosarcoma	6	1.2
Clear cell sarcoma	6	1.2
Angiosarcoma	5	1.0
Other	37	7.5
Benign lesions		
Lipomatous lesions/lipoma	41	8.3
Fibromatosis	37	7.5
Neurofibroma/schwannoma	33	6.7
Benign hyperplasia	27	5.4
Benign cystic lesion	23	4.6
Inflammatory lesions	21	4.2
Hemangioma	15	3.0
Nodular fasciitis	11	2.2
Giant cell tumor of the tendon sheath	6	1.2
Other	54	10.9

Abbreviation: UPS, undifferentiated pleomorphic sarcoma.

**TABLE 2:** FNA errors

Case ID	FNA diagnosis	Resection diagnosis
34	Negative for malignant cells	Alveolar soft part sarcoma
36	Negative for malignant cells	Myxofibrosarcoma
304	Negative for malignant cells	UPS
305	Negative for malignant cells	UPS
311	Negative for malignant cells	Myxofibrosarcoma
312	Negative for malignant cells	Fibrosarcoma
313	Negative for malignant cells	Myxofibrosarcoma
314	Negative for malignant cells	Well‐differentiated liposarcoma
315	Negative for malignant cells	Fibrosarcoma
316	Negative for malignant cells	Mucinous liposarcoma
317	Negative for malignant cells	UPS
318	Negative for malignant cells	Myxofibrosarcoma
328	Negative for malignant cells	UPS
329	Negative for malignant cells	Chondrosarcoma
330	Negative for malignant cells	Myxofibrosarcoma
331	Negative for malignant cells	Myxofibrosarcoma
335	Negative for malignant cells	UPS
336	Negative for malignant cells	Myxofibrosarcoma
340	Negative for malignant cells	Synovial sarcoma
341	Negative for malignant cells	UPS
342	Negative for malignant cells	Myxofibrosarcoma
343	Negative for malignant cells	Fibrosarcoma
344	Negative for malignant cells	Dedifferentiated liposarcoma
357	Negative for malignant cells	Mesenchymal tumor
358	Negative for malignant cells	Fibrosarcoma
359	Negative for malignant cells	UPS
360	Negative for malignant cells	Neuroendocrine neoplasm
363	Negative for malignant cells	UPS
368	Negative for malignant cells	Fibrosarcoma
369	Negative for malignant cells	UPS
370	Negative for malignant cells	Myxofibrosarcoma
371	Negative for malignant cells	Myxofibrosarcoma
372	Negative for malignant cells	Malignant schwannoma
373	Negative for malignant cells	Malignant schwannoma
374	Negative for malignant cells	Malignant schwannoma
376	Negative for malignant cells	Fibrosarcoma
377	Negative for malignant cells	UPS
387	Negative for malignant cells	UPS
389	Negative for malignant cells	Myxofibrosarcoma

Abbreviation: UPS: undifferentiated pleomorphic sarcoma.

A total of 164 cases (33.1%) could not be determined as being malignant or benign based on FNA (Table 3). These STL were suspected to be malignant (*n* = 84) or spindle cell present (*n* = 80) based on FNA. In the suspicious group, 69 of the 84 cases (82.1%) were malignant based on postoperative pathology. However, in the spindle cell group, the probability of malignancy declined, and 26 of the 80 cases (32.5%) were proven to be malignant. In the malignant (*n* = 90) and benign (*n* = 242) groups evaluated using FNA, the diagnostic sensitivity was 69.8%, specificity was 100%, PPV was 100%, NPV was 83.9%, accuracy was 88.3%, and Youden index was 69.8% (Table 4). In accordance with our results, the other seven available validation cohorts (*n* = 1157) also confirmed that FNA had a high rate of PPV (93.8%–100%, Table 5). As STL was negative for malignancy based on FNA, including suspicious for malignancy, spindle cell present, and benign lesions, FNA could not make an accurate diagnosis in identifying malignant or benign lesions; therefore, CNB is necessary for these patients after FNA.

**TABLE 3:** Summary of initial FNA and final postoperative diagnoses

Diagnostic method	Initial diagnosis	Final diagnosis		Total
		Malignancy	Benign	
FNA	Malignancy	90	0	90
	Suspicious for malignancy	69	15	84
	Spindle cell present	26	54	80
	Benign	39	203	242

**TABLE 4:** FNA for determining malignancy

	Accuracy	Sensitivity	Specificity	PPV	NPV	Youden index
FNA	88.3%	69.8%	100%	100%	83.9%	69.8%

**TABLE 5:** The PPV determined using FNA in our study and seven other validation cohorts

Study	Year	No. of cases	PPV (%)
Current study	2022	496	100
Wakely et al.^13^	2000	77	100
Rekhi et al.^18^	2007	115	98
Dey et al.^19^	2003	82	95.5
Fleshman et al.^20^	2007	91	97
Ng et al.^14^	2010	432	96.1
Beg et al.^21^	2012	126	97.2
Diaz et al.^22^	2018	234	93.8

Abbreviation: PPV: positive predictive value.

**FIGURE 2:**
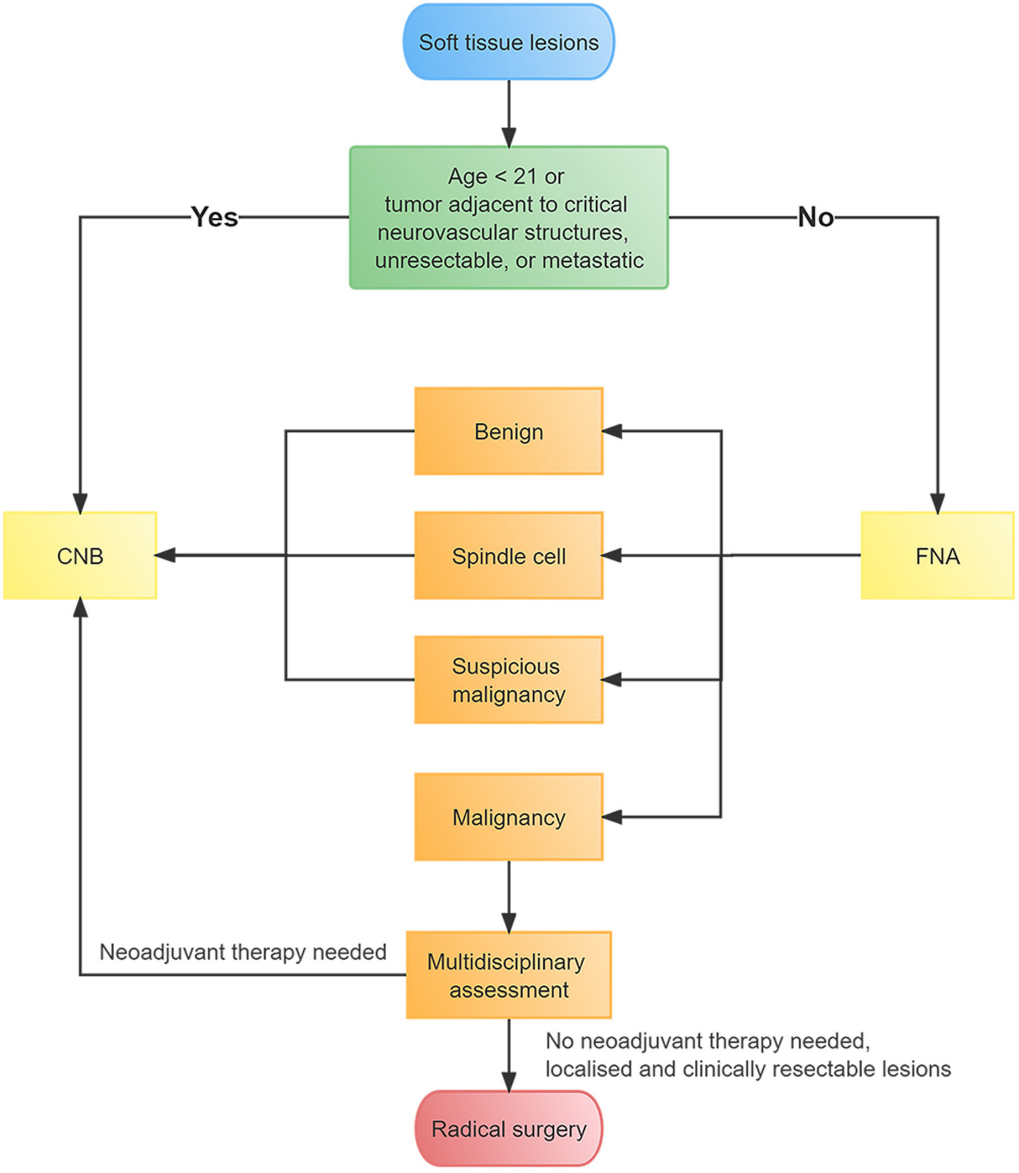
An innovative diagnostic flowchart for soft tissue lesions.

Previous studies have focused on the accuracy of biopsy in terms of STS pathological subtypes, but few studies have shown the capability of soft tissue FNA biopsy to guide surgical treatment. Considering that surgical resection (with appropriately negative margins) is the standard primary treatment for most patients with STS, according to the NCCN Guidelines,^8^ and our results show that FNA has a high PPV for detecting malignancy, we established an innovative diagnostic flowchart for STL, as shown in Figure 2. It is noteworthy that patients who are no more than 20 years of age have a high probability of pediatric sarcoma (such as rhabdomyosarcoma and Ewing sarcoma), and multidisciplinary evaluation and treatment planning are required.^8^ Therefore, patients under 21 years of age are recommended to undergo CNB. If the tumor is adjacent to critical neurovascular structures or the lesion is unresectable using radiologic evaluations or tumors with metastasis, patients should first undergo CNB to identify histological types without FNA. Otherwise, patients should first undergo FNA. If FNA reveals that the lesion is benign, spindle cells are present, or it is suspicious for malignant cells, the patients should undergo CNB. If FNA shows malignancy, a multidisciplinary assessment can indicate whether neoadjuvant therapy is needed, according to the medical conditions of the patients and the NCCN guidelines. If appropriate, radical surgical intervention will be performed without preoperative CNB (Figure 2). The value of the procedure is that CNB can be omitted for resectable STL with malignant FNA.

## 4 DISCUSSION

STS is a rare and heterogeneous disease with various subtypes. A biopsy is a key step in the diagnosis of primary musculoskeletal tumors. Biopsy methods include FNA, CNB, incisional biopsy, and excisional biopsy.^2^ To the best of our knowledge, the current study (*n* = 496) is one of the largest series of FNA biopsies of STL reported to date. We found that FNA has a unique advantage for detecting malignancy with PPV 100% (*n* = 496), which is consistent with seven other validation cohorts (PPV all more than 93%, *n* = 1157, Table 5). For patients with resectable lesions and malignant FNA, the CNB step can be omitted with a multidisciplinary assessment, and radical surgery can be performed. The value of this procedure is that CNB can be omitted with multidisciplinary evaluation for resectable lesions with malignant FNA.

CNB is a highly recommended technique for the diagnosis of primary STL. Previous reports indicate that the accuracy of CNB can be above 90%.^23^, ^24^, ^25^ Therefore, the NCCN guidelines recommend CNB as the first‐line treatment for the diagnosis of STL.^8^ However, because surgical resection (with appropriately negative margins) is the standard primary treatment for most patients with STS, according to the NCCN Guidelines,^8^ it is important to determine whether the lesion is malignant or benign before the surgery, which guides the excision scope. In our study, we compared the 496 FNA results with their corresponding postoperative histopathologies; 90 cases (18.2%) were positive for malignancy based on FNA and were also confirmed as malignant lesions through postoperative pathology. FNA showed a PPV of 100% in terms of malignancy. Consistent with our study, the other seven cohorts (Table 5, *n* = 1157) showed that FNA had high rates of PPV, which ranged from 93.8% to 100%. A meta‐analysis study also showed that FNA had a consistently high PPV, with value of 97.6% in STL.^26^ These results suggest that patients with malignant FNA are mostly diagnosed with malignant diseases based on postoperative pathology. Therefore, for patients with resectable lesions and malignant FNA, the CNB step can be omitted with multidisciplinary assessment, and radical surgery can be performed. To the best of our knowledge, this is the first study to show that FNA can guide STS surgery in treating STS.

Compared with CNB, FNA has many advantages, such as no need for local anesthesia, invasive trauma is minimal, diagnosis is rapid, and cost is lower.^13^, ^14^ Preparation of the pathological report usually requires more than 2 weeks for CNB; however, the report for FNA is ready after 2 h in our hospital. Furthermore, repeated immunohistochemistry should be performed on postoperative samples for patients who have undergone CNB, thus imposing additional financial burdens on these patients. However, a disadvantage of FNA is that the histologic subtyping of STS cannot be defined by FNA. If the lesion is shown to be unresectable or metastasizes using imaging examinations, surgery may not be the preferred treatment. Acquisition of pathological classification using CNB is a top priority, and a multidisciplinary approach utilizing surgery, chemotherapy, radiotherapy, immunotherapy, targeted therapy, and other treatments is essential for correctly assessing and treating STS. It is noteworthy that patients who are no more than 20 years of age have a high probability of pediatric sarcoma (such as rhabdomyosarcoma and Ewing sarcoma), and multidisciplinary evaluation and treatment planning are required for these patients.^8^ In our cancer center, STL are more common in adults. Only 9 patients under the age of 21 years were excluded from the present study. For patients under the age of 21 years, we first recommend CNB rather than FNA.

In this study, FNA yielded a high PPV for detecting malignant tumors. Of the 496 aspirates of FNA, 90 (18.2%) were positive for malignancy. Interestingly, FNA could correctly detect all of the 90 malignant lesions, according to postoperative pathology. However, the probability of malignancy gradually declined in the suspicious, spindle cell, and benign groups. This study showed that 69 of 84 cases (82.1%), 26 of 80 cases (32.5%), and 39 of 242 cases (16.1%) were proven to be malignant in the suspicious, spindle cell, and benign groups, respectively. Among the histologic subtypes, UPS and myxofibrosarcoma were most likely to be misdiagnosed as false negatives based on FNA, which may be explained by the cells of UPS being highly atypical^27^ and myxofibrosarcoma containing many myxoid substances^28^; secondly, the UPS and myxofibrosarcoma were the top two sarcoma subtypes in this study (Table 1); thirdly, malignant cells that could not be defined using FNA may be because of insufficient or paucicellular material, central necrosis, and non‐lesional material.^29^, ^30^ Regardless of whether the FNA is suspicious, spindle‐shaped, or benign, malignancy cannot be excluded. Therefore, CNB is recommended for patients without malignant FNA.

This study had some limitations. For patients with resectable lesions and malignant FNA, radical surgery may be performed without preoperative CNB; however, the pathological classification and grading of STL are unknown before surgery. We can overcome this shortcoming through the following: First, we exclude pediatric sarcoma and STL that are adjacent to critical neurovascular structures, as previously described. Second, there is no uniform use of neoadjuvant chemotherapy in resectable, localized STS, and a published clinical controlled trial has shown no benefit from neoadjuvant chemotherapy.^31^, ^32^ Third, radiotherapy may be added to the treatment of high‐grade (G2–3) lesions and is often delivered preoperatively.^32^ However, local control and overall survival (OS) are not influenced by the timing of radiotherapy.^33^, ^34^ Therefore, radiotherapy can be delivered in the postoperative setting according to postoperative histopathology, grading, and surgical margins. Fourth, we did not perform immunohistochemistry, cytogenetics, or molecular genetic testing after FNA, which may have improved the diagnostic accuracy. However, in practice, localized and resectable lesions can be rapidly determined using FNA. For malignant FNA, because surgical resection (with appropriately negative margins) is the standard primary treatment for most patients with STS, according to the NCCN Guidelines,^8^ the CNB step (including immunohistochemistry, cytogenetics, or molecular genetic testing) can be omitted, and radical surgery can be performed with multidisciplinary assessment. The innovative diagnostic flowchart shown in Figure 2 is convenient and inexpensive to use, decreasing waiting time in the treatment of resectable STL with malignant FNA. However, if neoadjuvant therapy is needed, as shown by multidisciplinary evaluation for STL with malignant FNA, CNB can be performed. Additionally, the diagnostic flowchart can be flexibly applied in developing countries and poor areas to advance diagnosis and treatment.

In conclusion, our study reviewed a large series of soft tissue FNA biopsies (*n* = 496) at the Fudan University Shanghai Cancer Center. Our results demonstrate that FNA has a high PPV for malignancy. The other seven validation cohorts (*n* = 1157) also supported this finding. We have established an innovative diagnostic flowchart for STL, as shown in Figure 2. For patients with resectable lesions and malignant FNA, the CNB step can be omitted with multidisciplinary assessment, and radical surgical intervention can be performed. In cases where FNA is negative for malignancy, patients should undergo CNB. The value of this procedure is that CNB can be omitted with multidisciplinary evaluation for resectable lesions with malignant FNA.

## AUTHOR CONTRIBUTIONS

B.Z. and W.Y. participated in the study design. B.Z. critically revised the manuscript. B.W. drafted the manuscript. F.M. analyzed the data. All authors have read and approved the final manuscript.

## FUNDING INFORMATION

This study was supported by grants from the National Natural Science Foundation of China (Grant No: 81302103).

## CONFLICT OF INTEREST

The authors declare no conflict of interests.

## Data Availability

N/A
